# Bone Single Photon Emission/Computed Tomography in the Detection of Sacroiliitis in Seronegative Spondyloarthritis: A Comparison with Magnetic Resonance Imaging

**DOI:** 10.4274/mirt.50570

**Published:** 2017-10-02

**Authors:** Theodoros Pipikos, Dimitrios Kassimos, George Angelidis, John Koutsikos

**Affiliations:** 1 401 General Military Hospital, Clinic of Nuclear Medicine, Athens, Greece; 2 401 General Military Hospital, Clinic of Rheumatology, Athens, Greece; 3 Army Share Fund Hospital (417 NIMTS), Clinic of Nuclear Medicine, Athens, Greece

**Keywords:** Bone scintigraphy, SPECT, Sacroiliitis, spondyloarthritis, magnetic resonance imaging

## Abstract

**Objective:**

Seronegative spondyloarthritis (SpA) is characterized by chronic inflammation affecting the axial skeleton, entheses and occasionally peripheral joints. The involvement of the sacroiliac joints, sacroiliitis, is considered as a pathognomonic radiographic finding. Magnetic resonance imaging (MRI) is the method of choice for its early detection. Bone scintigraphy (BS) is characterized by high sensitivity in the diagnosis of bone and articular diseases. Limited value of BS in the diagnosis of sacroiliitis may be attributed to the use of planar imaging. In the present study, we aimed to investigate the role of SPECT in SpA, compared to MRI.

**Methods:**

Forty-three patients suffering from inflammatory back pain underwent MRI evaluation of the sacroiliac joints and BS, combined with SPECT in the same region, for the assessment of sacroiliitis.

**Results:**

Bone SPECT revealed no findings of sacroiliitis in 11 patients, with total agreement with MRI. Findings of chronic lesions were demonstrated from both modalities in 2 patients. Bone SPECT and MRI findings were in concordance regarding the investigation of active sacroiliitis, with the exception of one patient with mild SPECT findings and negative MRI examination; the diagnosis of AS however, was established one year later, after a positive follow-up MRI. The evaluation of the planar imaging of the whole skeleton and SPECT imaging, revealed additional lesions.

**Conclusion:**

Bone SPECT is a reliable imaging method in the diagnosis of active sacroiliitis. Its application on planar BS, an economic and widely available diagnostic technique, appears to be a valuable aid for the clinician.

## Abstract (tr)

**Amaç:**

Seronegatif spondiloartrit (SpA) aksiyal iskelet, entezler ve nadiren perifer eklemlerin kronik enflamasyonu ile karakterizedir. Sakroiliak eklem tutulumu, sakroiliit, patognomonik bir radyografik bulgu olarak kabul edilir. Manyetik rezonans görüntüleme (MRG) erken tanı için tercih edilen yöntemdir. Kemik sintigrafisi (KS) kemik ve eklem hastalığının tanısında yüksek duyarlılığa sahiptir. KS’nin sakroiliit tanısındaki kısıtlı değeri planar görüntüleme kullanılmasına bağlı olabilir. Bu çalışmada SpA’da SPECT’in rolünün MRG ile karşılaştırılması amaçlandı.

**Yöntem:**

Enflamatuvar sırt ağrısı olan 43 hastaya sakroiliak değerlendirme için MRG ve KS uygulandı, sakroiliit tanısı için aynı bölgenin SPECT değerlendirilmesi ile kombine edildi.

**Bulgular:**

Kemik SPECT, MRG ile tam tutarlılıkla, 11 hastada herhangi bir sakroiliit bulgusu ortaya koymadı. İki hastada kronik lezyonlara ait bulgular her iki modalitede de görüldü. Kemik SPECT ve MRG bulguları aktif sakroiliit değerlendirmesi açısından hafif SPECT bulguları ve negatif MRG değerlendirmesi olan ancak bir yıl sonra pozitif takip MRG’si ile AS tanısı konulan bir hasta haricinde, tamamen uyumlu idi. Tüm iskelet sistemi planar görüntülemesi ve SPECT ile değerlendirme ek lezyonlar ortaya koydu.

**Sonuç:**

Kemik SPECT aktif sakroiliit tanısı için güvenilir bir görüntüleme yöntemidir. Ekonomik ve yaygın olarak erişilebilen bir tanı yöntemi olan planar KS ile kullanımı klinisyene yardımcı olabilecek değerli bir yöntem olarak gözükmektedir.

## INTRODUCTION

Ankylosing spondylitis (AS) is the most common form of seronegative spondyloarthritis (SpA). It is characterized by chronic inflammation affecting the axial skeleton, entheses and occasionally peripheral joints. The involvement of the sacroiliac joints, sacroiliitis, is considered as a pathognomonic radiographic finding, and is required for the diagnosis of AS based on the 1984 modified New York criteria (1). A broader term, axial spondyloarthritis (ax-SpA), has been introduced in order to describe all SpA types affecting the axial joints, including those without radiographic findings (2).

Magnetic resonance imaging (MRI) is the method of choice for the early detection of sacroiliitis (3). MRI can detect acute (and chronic) inflammation, even before the radiographic depiction of structural changes (erosions, sclerosis, ankylosis). In general, the interpretation of radiographic images regarding sacroiliitis is quite difficult, and the corresponding findings are present only after significant disease progression (4).

The role of single photon emission tomography (SPECT) imaging, as a sensitive and specific test for the detection of sacroiliitis and spinal inflammation, has been investigated previously (5). However, studies comparing SPECT and MRI are limited. In the present study, we aimed to investigate the role of SPECT in ax-SpA. Sacroiliac joints are formed by the articular surfaces of the sacrum and ilium; the upper third is a syndesmosis, whereas the other two thirds are lined by articular cartilage and only the lower third is lined by synovium. We proposed a scintigraphic classification based on the presence (or not) of intense focal radiopharmaceutical uptake at the synovial part, as a sign of active sacroiliitis. Then, we evaluated SPECT results in comparison to MRI findings.

## MATERIALS AND METHODS

The present study was conducted by the Department of Nuclear Medicine of the 401 General Military Hospital in Athens, Greece. Forty-three patients (38 males, 5 females, mean age 28.2 years) suffering from inflammatory back pain (IBP) with disease duration >6 months underwent MRI evaluation of the sacroiliac joints, and bone scintigraphy (whole body scan, combined with SPECT in the same region) for the assessment of sacroiliitis. Ten patients had IBP without established diagnosis of SpA, whereas 33 patients were diagnosed with SpA (22 patients with AS, 8 patients with undifferentiated SpA, 3 patients with psoriatic arthritis). Before testing, all subjects gave informed consent for their complete participation, in compliance with the Hospital Ethics Committee guidelines and the ethical guidelines of the 1964 Declaration of Helsinki. The study was approved by the Local Ethics Committee of the 401 General Military Hospital of Athens, Greece (Protocol number: 2009/5).

Radiographic stage was recorded in all patients according to the 1984 modified New York criteria (1). Absence of radiographic findings was apparent in 11 patients. Suspicious changes (stage 1) were observed in 11 patients, whereas 10 patients had minimal abnormalities (such as erosions or sclerosis) without alteration of joint width (stage 2). Eight patients were classified as stage 3 (unequivocal abnormalities with 1 or more of the following findings: erosions, sclerosis, widening, narrowing, or partial ankylosis), and total ankylosis was demonstrated in two patients (stage 4). In MRI studies, the presence of clear bone marrow edema on STIR images or osteitis on T1 post-gadolinium images was indicative of active sacroiliitis (new Assessment of Spondyloarthritis-ASAS-classification criteria) (2,6,7). Structural abnormalities (i.e. fat deposition, erosions, sclerosis, ankylosis) were associated with chronic disease in the absence of the aforementioned ASAS criteria.

Bone SPECT imaging was performed with the patient in prone position using a single head camera (Sophy ds7, France). Acquisition included 64 projections (45 sec/frame, 64x64 matrix) in an 180^0^ contour. Raw data was processed on a Mirage-Segami workstation based on iterative reconstruction methodology (RESPECT).

All participants underwent laboratory investigations that included measurements of erythrocyte sedimentation rate (ESR) and C-reactive protein (CRP), and the presence of human leukocyte antigen (HLA). The 2-tailed Mann-Whitney U nonparametric test was used to evaluate differences. Kruskal-Wallis test was used to assess differences in HLA. P values 0.05 were considered statistically significant.

Both imaging techniques, whole body scan and MRI, were performed within a seven day period, and datasets were evaluated by two blinded reviewers; a radiologist and a nuclear medicine physician with expertise in the imaging of the musculoskeletal system.

## RESULTS

### Bone SPECT Findings

**Figure 1:**
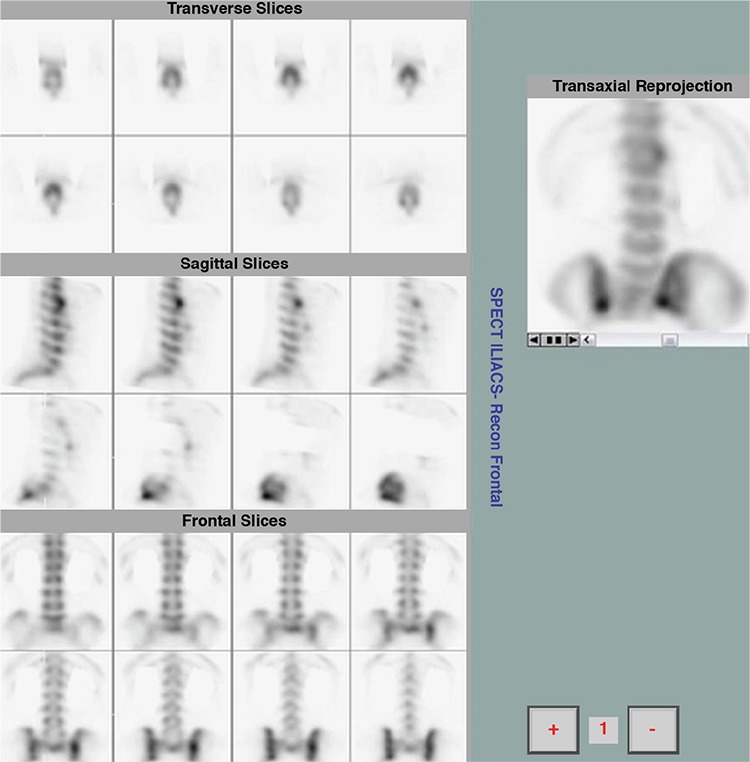
Intense focal uptake of the radiotracer at the base of the sacroiliac joints (synovial part), indicating the presence of active sacroiliitis (positive study). Also, enthesopathy of the T12-L1 vertebrae is noted (syndesmophytosis)

**Figure 2:**
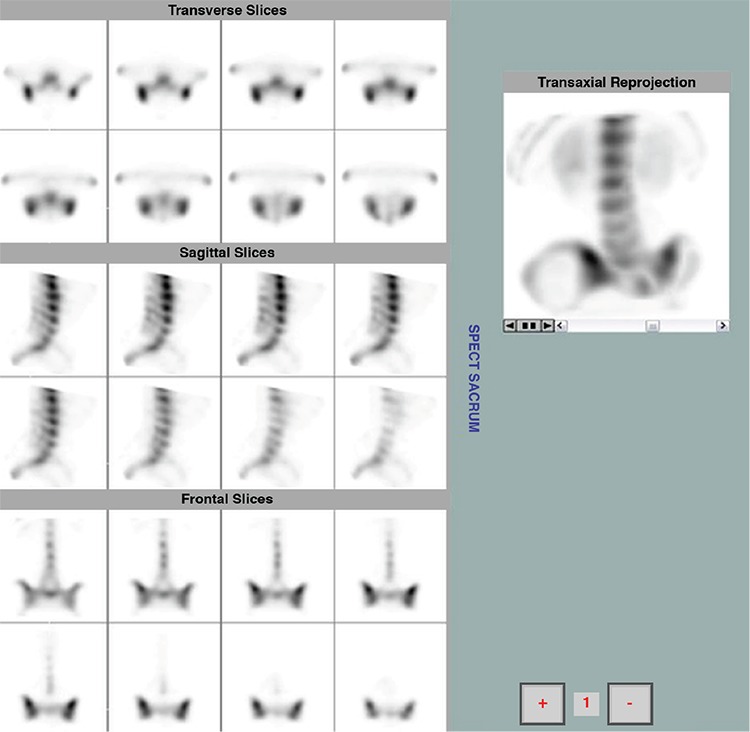
Absence of focal radiopharmaceutical uptake at the lower part of the sacroiliac joints (synovial part), excluding the presence of active sacroiliitis in a patient with negative magnetic resonance imaging findings

**Figure 3:**
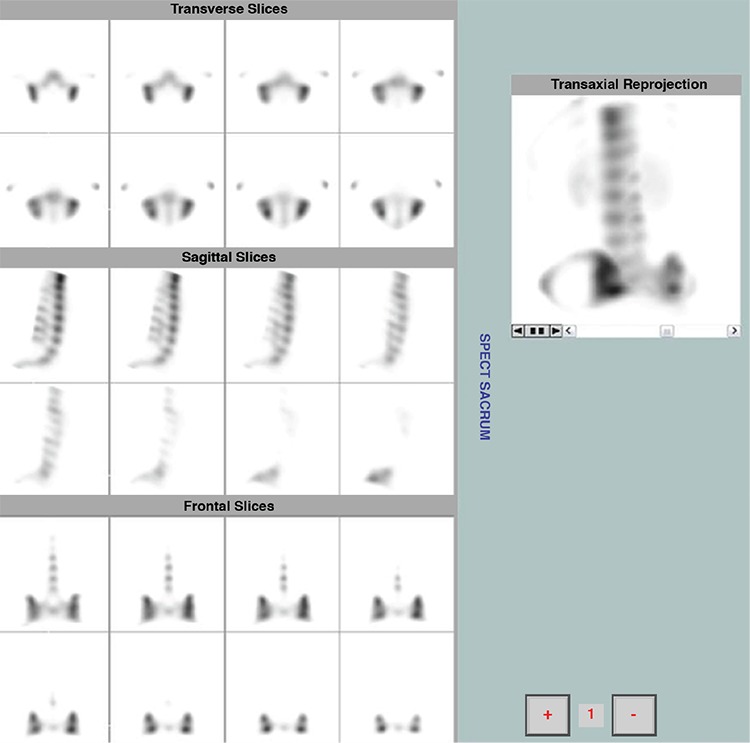
Diffuse increase in radiopharmaceutical uptake at the sacroiliac joints as a sign of chronic inflammation, co-existing with active disease

Bone SPECT images were classified as positive for active sacroiliitis (Figure 1) in patients with intense focal radiopharmaceutical uptake at the base of the sacroiliac joints (synovial part). Findings of active sacroiliitis were recorded in 30 patients; 23 patients with definite findings of active sacroiliitis (Table 1) and seven patients with mildly increased radiotracer uptake at the sacroiliac joints indicating low-grade sacroiliitis (Table 2). On the other hand, no uptake at the synovial part was indicative of a negative study (Figure 2). Bone SPECT revealed no findings of sacroiliitis in eleven patients. Finally, a diffused uptake at the sacroiliac joints was considered as a sign of chronic inflammation (Figure 3). Chronic sacroiliitis was found in two patients.

### Comparison with MRI and Radiographic Staging

In patients with no scintigraphic findings of sacroiliitis (stage 0: 9 patients, stage 1: 1 patient, stage 2: 1 patient), MRI was also negative. Moreover, concordant magnetic resonance imaging results were obtained in patients with scintigraphic findings of chronic sacroiliitis (stage 2: 1 patient, stage 4: 1 patient).

Among patients with definite scintigraphic findings of active sacroiliitis (stage 0: 1 patient, stage 1: 5 patients, stage 2: 8 patients, stage 3: 7 patients, stage 4: 2 patients), chronic inflammation co-existed with SPECT findings of active disease in 11 patients. MRI chronic structural changes were evident in three more patients. Two patients showed definite evidence of the disease in one sacroiliac joint, according to both imaging modalities.

Among patients with mildly increased radiotracer uptake at the sacroiliac joints (stage 0: 1 patient, stage 1: 5 patients, stage 2: 1 patient), six patients had mild findings of bone marrow edema in MRI without corresponding osteitis. However, no structural MRI abnormalities were found in one patient (stage 1) suffering from persistent symptoms. Interestingly, the presence of sacroiliitis was evident in the MRI study performed after one year, and the diagnosis of AS was eventually established.

### Whole Body Imaging

After evaluating planar images of the whole skeleton and SPECT findings of the lumbar spine-pelvis region, additional lesions were recorded (Table 3).

### Laboratory Variables

The mean values [± standard deviation (SD)] of ESR were 28.8±26.9 mm/hr in patients with active sacroiliitis (according to SPECT and MRI findings), 20.3±16.4 mm/hr in patients with findings of low-grade sacroiliitis, and 19.8±17.6 mm/hr in the remaining patients (P=NS). The mean values (± SD) of CRP were 20.2±16.7 mg/L, 15±14.1 mg/L and 36±37 mg/L, respectively (P=NS).

No correlations were found between HLA results and imaging findings according to bone SPECT and MRI.

## DISCUSSION

To the best of our knowledge, this is one of limited studies investigating the role of SPECT imaging in SpA. Our study population consisted of 43 subjects with a relatively low mean age (28.2 years). Also, we enrolled more males than females (38 vs. 5). These particularities of our study population are associated with the special role of our institution providing care mainly to men and women serving at the Hellenic Armed Forces. We showed that SPECT findings are reliable; eleven negative SPECT and MRI studies, positive SPECT findings with concordant MRI results in 31 patients, while in one patient with SPECT evidence of sacroiliitis and negative MRI, the diagnosis of AS was established in a later follow-up MRI study.

Traditionally, the diagnostic investigation and classification of SpA were based on plain radiograms of the sacroiliac joints. MRI was added to the diagnostic algorithm with the introduction of ASAS criteria (2,6,7,8). MRI findings of active sacroiliitis include bone marrow edema, capsulitis, synovitis and inflammation in tendons and ligaments. Particularly, bone marrow edema may be present in a number of pathological states; therefore, it is not a specific finding. On the other hand, synovitis and capsulitis are more specific. Furthermore, sclerosis, erosions, bony bridges and bone marrow transformation are considered as signs of chronic lesions. The inter-observer agreement in the assessment of inflammation in patients with IBP has been investigated in previous MRI studies showing adequate levels of agreement, but not exceeding 85% (9,10,11).

Nuclear medicine techniques, particularly bone scintigraphy, are characterized by high sensitivity in the diagnosis of bone and articular diseases, as well as enthesopathies (12). Initial trials investigating the role of bone scintigraphy in sacroiliitis assessment included planar imaging and a semi-quantitative approach. Regions of interest were drawn over the sacroiliac joints and reference structures, such as the sacrum and lumbar vertebrae (13). Although the results were promising, the diagnostic performance was inferior to MRI (14). On the other hand, preliminary bone SPECT studies confirmed the ability of the method for detecting sacroiliitis, indicating its potential role in the diagnostic investigation of these patients (15). The sensitivity and specificity of bone SPECT were demonstrated in a study that enrolled age-matched controls; the authors reported sensitivity of 85% and specificity of 90% in the detection of sacroiliitis (5). Ryan et al. (16) noted that SPECT abnormalities in the lower thoracic and lumbar spine are often observed in patients with AS, particularly in chronic cases. However, Çevik et al. (17) concluded that MRI provides the strongest evidence of the disease in cases showing clinical features of inflammatory lesions in the spine. More recently, Zilber et al. (18) suggested that bone SPECT combined with calculation of indices or low-dose computed tomography (CT) can be used in the diagnostic investigation of axial SpA, with favourable sensitivity and specificity. Interestingly, Cui et al. (19) reported that the diagnostic value of SPECT and MRI is comparable to that of plain radiograms and CT, and can be performed for the (quantification of the inflammatory process. Moreover, sacroiliac index measurements confirming positive findings may increase the specificity of bone scintigraphy (20). In our study population, bone SPECT and MRI findings were in concordance regarding the investigation of active sacroiliitis, with the exception of one patient with mild SPECT findings and negative MRI examination. However, the diagnosis of AS was established for this patient one year later, after a positive follow-up MRI. Further, MRI demonstrated chronic inflammation in more patients compared to bone SPECT, as expected based on the superior diagnostic performance of MRI in depicting chronic structural lesions.

In general, the previously observed limited value of bone scintigraphy in the diagnosis of sacroiliitis may be attributed to the use of planar imaging. Based on a systematic literature research that resulted in the analysis of 25 published studies, bone scintigraphy demonstrated overall sensitivity of approximately 50% in detecting sacroiliitis, either in patients with established AS or in patients with probable sacroiliitis (21). The interpretation of planar imaging findings in areas with complex anatomical features, such as the sacroiliac joints, could be quite challenging due to the overlying and underlying structures that may lead to false positive or false negative results. The limited diagnostic value of planar imaging (combined with semi-quantitative measurements) was also confirmed in a previous study performed in our institution. MRI detected features of active sacroiliitis in 34/36 patients, bone SPECT was in total agreement with MRI findings, whereas planar sacroiliac scintigraphy was positive in only 19/36 patients (22).

Significant advances in the field of SPECT imaging have been achieved during the previous decade. Particularly, the introduction of iterative reconstruction algorithms offered images of superior quality compared to those produced from other processing methods. Moreover, reconstructed 3D images permitted a more accurate evaluation of the synovial part of sacroiliac joints. Previously, due to the small dimensions of the synovial part with respect to the spatial resolution of SPECT technique, uptake in the sacrum or other parts of the sacroiliac joints could be falsely diagnosed as sacroiliitis. Given the current availability of 3D images, the clear discrimination of the lower synovial part facilitates the evaluation of the sacroiliac region, increasing intra-observer agreement regarding the diagnosis of the disease. However, at the time that the aforementioned studies investigated the role of SPECT imaging in the detection of sacroiliitis, these advances were not available.

Further, it is unclear if the diagnosis of sacroiliitis, in previous SPECT studies, was only linked to increased uptake at the synovial part (chronic changes associated with SpA can result in faint uptake at the whole joint mimicking active sacroiliitis). We suggest that active sacroiliitis should be reported only in cases with focal uptake at the synovial part; other types of uptake seem to be associated with chronic changes or enthesopathy. According to the results of the present study, focal uptake at the synovial part was demonstrated in 23/42 patients; therefore, SPECT findings were in accordance with the diagnosis of acute sacroiliitis based on MRI criteria. Moreover, the level of uptake in SPECT imaging was positively related to MRI features. Patients with higher uptake in bone scintigraphy showed more intense MRI findings. Among the 23 patients with active sacroiliitis based on MRI results, fourteen had also findings of chronic structural changes. 11/14 patients showed faint linear uptake at the sacroiliac joints which was associated with the co-existing chronic changes. However, 3D images permitted the depiction of focal uptake at the synovial part, enhancing the diagnostic performance of the technique. In 12 patients with no signs of the disease according to MRI technique, normal uptake of radiopharmaceutical was observed in the lower part of the sacroiliac joints in eleven patients. In two of these patients, low back pain was linked to the presence of facet arthritis located at the lumbar spine. Diffuse pattern of uptake (without focal uptake in the lower part of the sacroiliac joints) was demonstrated in two patients with MRI findings indicating chronic changes. Additional lesions, including spinal lesions, peripheral arthritis and enthesitis, were revealed after the evaluation of the whole skeleton, while we suggest that if a SPECT study of thoracic spine, or a three phase scan were added, further findings could be demonstrated.

SPECT imaging was performed with a 180-degree rotation of the gantry, a procedure that increased the counts collected from the spine in a reasonable time frame with no further radiation for the patients compared to the whole body scintigraphy. Notably, the radiation burden of the whole procedure is not too high, with a total effective dose of approximately 5 mSv (for example, a plain x-ray of the lumbar spine is associated with an effective dose of 1.5 mSv). Obviously, it is linked to the limitations related to radiation exposure (e.g. pregnant women, patients who underwent multiple radiological examinations in a short time period). Therefore, the diagnostic evaluation, as described above, can be performed as a one-stop examination, providing valuable evidence concerning both the axial and the appendicular skeleton. Further studies could also investigate the use of bone SPECT as an imaging tool for the assessment of treatment outcomes in patients with SpA. In the same field, Zilber et al. (18) have proposed a potential role of 18F positron emission tomography and immunoscintigraphy with labelled monoclonal anti-cytokine antibodies in the evaluation of patients with suspected sacroiliitis.

## CONCLUSIONS

In conclusion, our study, based on imaging findings in patients with SpA, demonstrated the potential role of bone SPECT in the diagnostic investigation of SpA. According to our results, bone SPECT is a reliable imaging method in the diagnosis of active sacroiliitis and enthesitis. In comparison to MRI, whole body scan combined with SPECT imaging is a less expensive diagnostic approach per patient, while it is more widely available. Nevertheless, the implementation of this procedure in the clinical setting will require proper adjustment of the diagnostic investigation in patients with suspected sacroiliitis. Moreover, larger multi-centre studies are needed to confirm these encouraging findings regarding the role of bone SPECT in the detection of sacroiliitis in patients with SpA.

## Figures and Tables

**Table 1:**
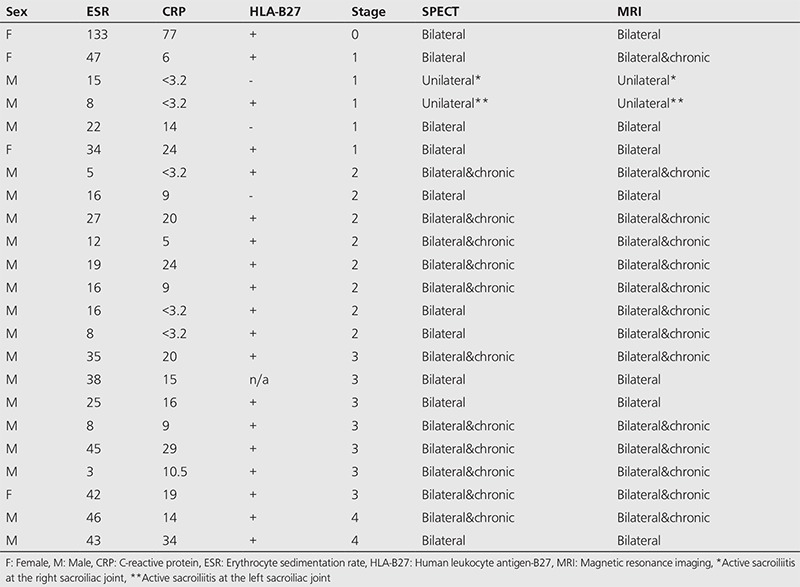
Patients with definite findings of active sacroiliitis

**Table 2:**
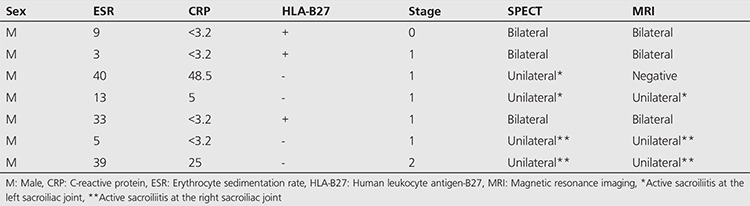
Patients with mildly increased radiotracer uptake at the sacroiliac joints, indicating low-grade sacroiliitis

**Table 3:**
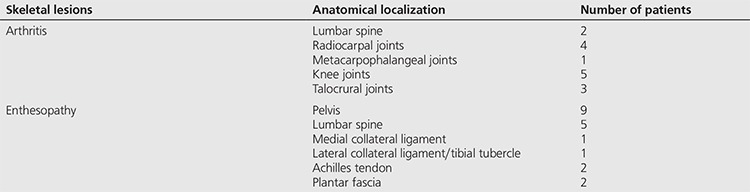
Additional lesions revealed based on the evaluation of the whole skeleton
